# Coronary artery disease mimicking Tako-tsubo cardiomyopathy: a case report

**DOI:** 10.4076/1757-1626-2-6374

**Published:** 2009-08-06

**Authors:** Ibrahim Halil Kurt

**Affiliations:** Department of Cardiology, Adana Numune Education and Research HospitalAdanaTurkey

## Abstract

Tako-tsubo cardiomyopathy is a syndrome mostly observed in post-menopausal women, which mimics myocardial infarction with an ST elevation; and coronary angiography shows apical ballooning but a normal left anterior descending. Although coronary artery disease is considered as an exclusion criterion, for differential diagnosis of this type of cardiomyopathy, Tako-tsubo cardiomyopathy cases accompanied by coronary artery disease were also reported. In this report, we are presenting a patient who exhibits different findings than a classical Tako-tsubo cardiomyopathy case because of her young age, coexisting atherosclerotic lesion, smaller diameter of the apical systolic ballooning and absence of an increase in cardiac enzymes.

## Introduction

Tako-tsubo cardiomyopathy (TTC) was first described as a case series of five patients by Dote *et al.* in 1991 [1]. TTC is also known as stress-induced or ampulla cardiomyopathy [2]. It is a clinical syndrome characterized by typical chest pain following emotional and physical stress, a typical ST segment elevation on ECG, mild increase in cardiac enzymes, and reversible systolic ballooning in the left ventricular apical region without any abnormality in coronary arteries. Although presence of atherosclerotic coronary artery disease is considered as an exclusion criterion for TTC diagnosis, TTC cases accompanied by coronary artery disease were also reported [3]. Our case presented in this report had a small-diameter systolic ballooning in the left ventricular apical region, a normal left anterior descending (LAD), but a severe atherosclerotic lesion on RCA.

## Case presentation

A 35-year-old Caucasian Turkish female patient presented to the emergency ward due to a sensation of burning pain in her chest. The patient was admitted to the coronary intensive care unit with the preliminary diagnosis of acute coronary syndrome.

**Figure 1.**
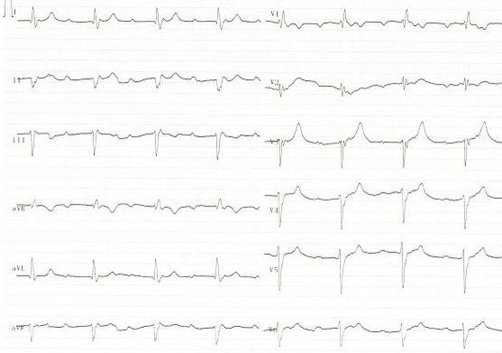
The ECG trace obtained at the emergency room showing an ST segment elevation on anterior leads (V1-3).

For the last 3 months, she had increasing emotional stress and complaints of a burning pain in her chest spreading to her left arm and back upon exercise. Her medical history revealed no established coronary risk factors. Upon physical examination, arterial blood pressure was 100/70 mmHg, pulse was 80 beats/min; and the other findings considered as normal. ECG performed at the emergency ward detected an ST segment elevation on the anterior leads (V1-3) (Figure 1). The patient was administered 300 mg acetylsalicylic acid (ASA), 5 mg sublingual isosorbide dinitrate and 5 mg intravenous morphine at the emergency ward. The cardiac markers were as follows: creatine kinase isoenzyme MB (CK-MB) 2.2 ng/ml (0.0-4.3), myoglobin 53.3 ng/ml (0.0-107) and cardiac troponin I (cTnI) 0.3 ng/ml (0.0-0.4).

**Figure 2.**
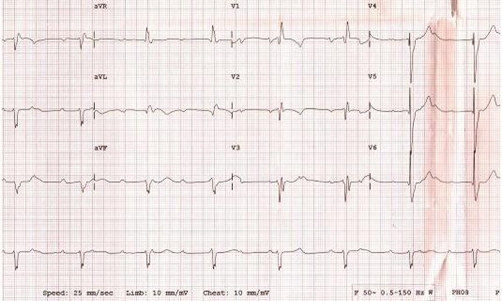
The ECG trace obtained after her chest pain relieved, showed a rapid resolution in the previously elevated ST segment.

**Figure 3.**
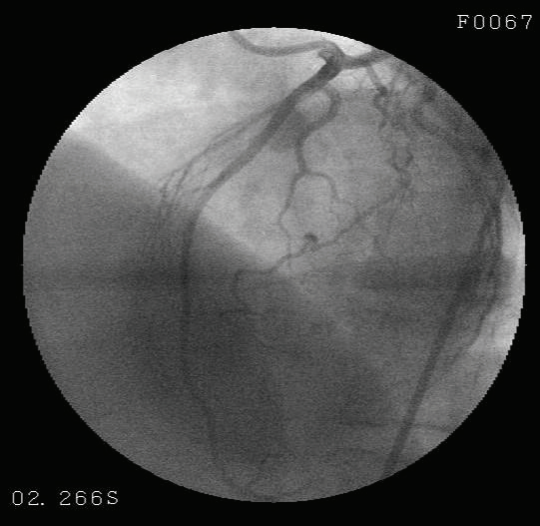
Coronary angiography revealed a normal LAD.

**Figure 4.**
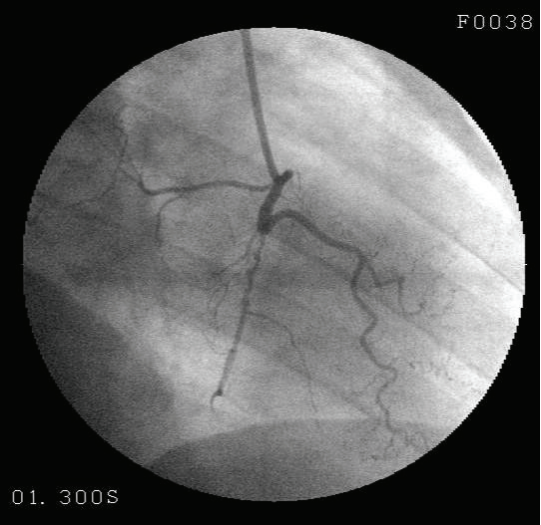
A rudimentary RCA was detected to exhibit a 95% occlusion following the right ventricular branch.

**Figure 5.**
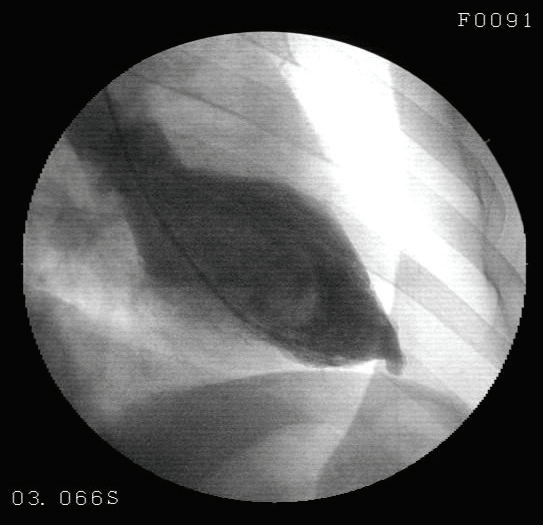
Left ventriculography showed a small ballooning in the apical region expanding outwards during systole.

When the patient was admitted to the coronary intensive care unit, her chest pain resolved and the follow-up ECG detected rapid resolution in the previously elevated ST segment (Figure 2). On the second day, cardiac markers were as follows: CK-MB 1.2 ng/ml (0.0-4.3), myoglobin 65.4 ng/ml (0.0-107), and cTnI 0.037 ng/ml (0.0-0.4). As for the other biochemical parameters, total cholesterol was 126 mg/dL, HDL-cholesterol 30 mg/dL, LDL-cholesterol 66 mg/dL, C-reactive protein (CRP) 1.1 (0-5) mg/L, homocysteine 1.38 µg/ml (0.5-2.2), and erythrocyte sedimentation rate 38mm/hour. Coagulation factors (Antithrombin III 138.15 (63-122%), Protein C 126.88 (78-134%), Protein S 54.44 (55-160%), Factor V Leiden (FVL) anti-phospholipid antibody, antinuclear antibody (ANA) and endocrinologic measurements were within normal limits. Upon transthoracic echocardiography all findings were normal except hypokinetic, apicoseptal wall. Coronary angiography revealed a normal LAD and circumflex (Cx) (Figure 3), while RCA was rudimentary and 95% occluded following right ventricular branch (Figure 4). Left ventriculography showed a hypokinetic inferior wall and minor ballooning in the apical region expanding outwards during systole (Figure 5). Myocardial perfusion scintigraphy was performed a week later, and detected a perfusion defect on the antero-apical wall. The patient was prescribed ticlopidin 250 mg twice daily, ASA 300 mg once daily, ramipril 5 mg once daily and metoprolol 50 mg once daily, and discharged.

## Discussion

Tako-tsubo cardiomyopathy (TTC) frequently manifests as chest pain and dyspnea following emotional and physical stress in post-menopausal women (90%) [4]. It is a clinical syndrome characterized by ST segment elevation on ECG, and transient ballooning in the apex together with normal epicardial coronary arteries on coronary angiography. A very small portion of cases with TTC involves young females [5]. Although the reason is not exactly established, the potential protective role of sex hormones against myocardial stunning is suggested [6].

Since cases with TTC mimic myocardial infarction with ST segment elevation, differential diagnosis is initially quite difficult. This type of cardiomyopathy is observed in 0.7-2.5% of acute coronary syndrome cases [7] and 0.3% of those underwent angiography [8]. Although the exact cause of TTC is not known, coronary vasospasm [9], coronary microcirculation abnormalities [10,11] and catecholamine-mediated cardiotoxicity are considered to be involved in the pathogenesis [4]. Significant stenosis is not observed in the epicardial coronary arteries in this syndrome [12]. Coronary stenosis < 50% in 19.04% of the cases on coronary angiography was detected in 10 different clinical trials [13].

TTC is typically localized in the middle and apical region of the left ventricle. Apical ballooning is usually reversible and benign. Although rare, severe complications such as cardiogenic shock due to mitral papillary muscle and myocardial free wall rupture may be observed in some cases.

Presence of atherosclerotic coronary stenosis is defined as an exclusion criterion in TTC diagnosis. However, there are some publications reporting that patients with coronary artery disease may also develop TTC [3]. Furthermore, in another trial, TTC cases were detected to have concomitant classical cardiovascular risk factors (diabetes, smoking habit, dyslipidemia and hypertension in 11%, 23%, 25% and 43% of the patients, respectively) [13]. Our case had no coronary risk factors in her history except stress; who was complaining about a typical chest pain and had ST segment elevation, systolic ballooning in the left ventricular apical region and angiographically normal LAD. The diameter of the systolic ballooning in the apex was small in our patient. Indeed, the size of apical ballooning in TTC is not among the diagnostic criteria for TTC [14]. We believe that the coexistence of severe stenosis of right coronary artery and small apical left ventricular aneurysm is coincidental.

Although mild increase may be seen in cardiac enzymes in TTC, no such increase was detected in our case on serial follow-ups. In a systemic review of 14 trials, 81.6% of the cases were detected to have ST segment elevation, 64.3% of the patients were detected to have Q wave and T wave abnormalities, and 86.2% of the cases exhibited a mild increase in cardiac biomarkers [13]. The rapid relieve of pain and a parallel spontaneous ST segment resolution within a very short period of time is another noticeable point.

## Conclusion

Although there is no evidence that atherosclerotic process is involved in the pathogenesis of TTC, accompanying coronary risk factors have been reported in TTC cases. While these two phenomena occur via different mechanisms, they may coexist. Therefore, atherosclerotic coronary lesions may be present in the other coronary arteries that are not involved in apical ballooning at the time of TTC diagnosis. Our patient exhibits findings different from classical TTC description that are young age, coexistence of atherosclerotic lesion, small diameter of apical systolic ballooning and absence of increase in cardiac enzymes.

## Abbreviations

ANA: antinuclear antibody
ASA: acetylsalicylic acid
CK-MB: creatine kinase isoenzyme MB
CRP: C-reactive protein
CTnI: cardiac troponin I
Cx: circumflex
ECG: electrocardiography
FVL: Factor V Leiden
LAD: left anterior descending
RCA: right coronary artery
TTC: Tako-tsubo cardiomyopathy
